# Specific microRNAs are associated with fracture healing phases, patient age and multi-trauma

**DOI:** 10.1016/j.jot.2022.07.002

**Published:** 2022-08-31

**Authors:** Rald Victor Maria Groven, Carlos Julio Peniche Silva, Elizabeth Rosado Balmayor, Bart Nicolaas Jacobus van der Horst, Martijn Poeze, Taco Johan Blokhuis, Martijn van Griensven

**Affiliations:** a Department of Cell Biology-Inspired Tissue Engineering, MERLN Institute for Technology-Inspired Regenerative Medicine, Maastricht University, Universiteitssingel 40, 6229 ER Maastricht, the Netherlands; b Division of Traumasurgery, Department of Surgery, Maastricht University Medical Center, P. Debyelaan 25, 6229 HX Maastricht, the Netherlands; c Department of Orthopaedic, Trauma, and Reconstructive Surgery, RWTH Aachen University Hospital, Pauwelsstraße 30, 52074 Aachen, Germany; d NUTRIM, School for Nutrition and Translational Research in Metabolism, Maastricht University, Universiteitssingel 40, 6229 ER Maastricht, the Netherlands

**Keywords:** Clinical parameters, Fracture healing, Gene expression, microRNAs, miRNA target analysis

## Abstract

**Background:**

Immediately after a fracture occurs, a fracture hematoma (fxH) is formed. This fxH plays an important role in fracture healing and, under normal circumstances, aids in generating an environment in which a wide variety of cells orchestrate processes involved in fracture healing. MicroRNAs (miRNAs) may influence these processes. The aim of this study was therefore to determine the miRNA expression signature of human fxH in normal fracture healing and examine the potential influence of clinical parameters on these expression levels.

**Methods:**

fxH was harvested from 61 patients *(mean age 52 ± 19; 32*♀*)* during fracture surgery. miRNAs were isolated, transcribed and pooled for qPCR array analysis and validation. Qiagen fibrosis- and inflammation qPCR arrays were used based on an extensive literature study related to fracture healing and osteogenesis. Additionally, miRNA targets were determined.

**Results:**

From the array data, a selection of the twenty most regulated miRNAs, 10 up- and 10 down regulated, was validated in the study population. The expression levels of seven out of these twenty miRNAs were correlated to several clinical parameters. The time interval between trauma and surgery showed to influence the expression of three miRNAs, three other miRNAs were expressed in a patient age dependent manner and one miRNA was associated with the severity of trauma.

**Conclusion:**

This study portrayed the role and importance of miRNAs in human fxH, linked to key processes in fracture healing. Seven miRNAs showed to be involved in multiple processes that are important in the fracture healing cascade, such as angiogenesis, mineralisation and cellular differentiation. *In silico* target analysis revealed 260 mRNA targets for 14 out of the 20 validated miRNAs.

**The Translational Potential of this Article:**

These data broaden our view on the potential diagnostic and therapeutic implications of miRNAs in fracture healing.

## 1 Introduction

Fracture healing is a complex, dynamic process that involves various cell types and cellular communication mechanisms. Physiological fracture healing can be divided into four main phases: the inflammatory, soft callus, hard callus, and remodelling phases [1]. The fracture hematoma (fxH) plays a central role during the first phase of healing. Studies have shown that removal or debridement of the fxH can negatively affect fracture healing [2]. A balanced inflammatory response seems to be of great importance since an exacerbated inflammatory response can disrupt physiological fracture healing [1]. During the inflammatory phase, a multitude of pro-inflammatory and angiogenic cytokines is released in the fracture zone to enhance chemotaxis and initiate angiogenesis. The latter is particularly important to supply the fracture zone with nutrients, cells and signalling molecules, but also to normalise the acidic and hypoxic conditions which are created due to the lack of proper vascularization after trauma. A variety of immune cells infiltrates the fxH, and a microenvironment is created in which the first steps towards the second phase of fracture healing, the bone repair phase, can be made [1,2].

During the soft and hard callus phases, a more regenerative microenvironment is required for fracture healing to take place, in which chondrocytes and osteoblasts form a granulation tissue called callus. Collagen formation and mineralisation of the granulation tissue are among important cellular processes in normal fracture healing. As time progresses, the callus gets ossified and the fracture gap is bridged. This process depends on the adequate supply of nutrients and cellular activity, which greatly relies on angiogenesis [1].

Failing angiogenesis, or poor vascularization in the tissues surrounding the fracture can therefore also result in impaired fracture healing [3]. The amounts of osteoclasts and osteoblasts at the fracture zone increase upon enhanced vascularization, enabling the formation of woven bone. The fourth phase of fracture healing, remodelling, lasts up to several months in which remaining callus is resorbed and the woven bone is replaced with lamellar bone [1].

Disturbance of the multiphase fracture healing process can result in fracture healing impairments such as delayed unions or non-unions [4]. These fracture healing impairments can elicit invalidating consequences for patients, and treatment often consists of multiple surgical interventions followed by long rehabilitation periods. Unfortunately, even though risk factors for the development of fracture healing impairments have been identified, the exact molecular mechanisms behind their development are poorly studied [4]. A first step in better understanding the pathophysiology of fracture healing impairments would be to investigate the cellular communication and molecular regulation of normal fracture healing.

MicroRNAs (miRNAs) are small, non-coding RNA molecules consisting of approximately 22 nucleotides. They are important post-transcriptional regulators that play key roles in both inter- and intracellular signalling [5]. MiRNAs have been extensively researched in the fields of oncology and endocrinology [6,7]. Regarding bone regeneration, *in vitro* studies have shown that miRNAs can influence processes that are involved in fracture healing, such as osteogenesis, angiogenesis, and inflammation [8,9]. *In vivo* work by Seeliger et al. revealed 5 differentially expressed miRNAs linked to osteoporotic fractures in human serum- and bone tissue samples [10]. In addition, miRNAs have shown to play a role in multiple musculoskeletal disease mechanisms [11]. For example, miRNA-21-5p and miRNA-216a-5p have shown to influence inflammation, osteogenesis, and chondrocyte migration [[12], [13], [14]]. Furthermore, miRNA-21-5p has shown to be involved in osteoporosis [10]. In a study conducted by Kelch et al. the authors showed both that miRNA-21-5p was upregulated in serum, bone tissue, osteoblasts, and osteoclasts of osteoporotic patients, and confirmed a correlation between expression of this miRNA and bone mineral density. The study by Kelch et al., along with existing literature, demonstrated the potential of miRNAs as target for therapeutic inhibition, as well as biomarkers in osteoporosis and other bone diseases [[13], [15]]. Although some studies have been conducted on the role of miRNAs in fracture healing, research connecting these to the specific phases of healing in humans is lacking.

To the best of our knowledge, no research has been done on miRNA expression in human fxH. Given the association of miRNAs with bone regeneration, it is important to investigate the miRNA expression and its regulation in human fxH and analyse the role of these miRNAs in the different processes within the fracture healing cascade. In particular, the influence of patient characteristics and clinical parameters on miRNA expression must be evaluated to assess the potential clinical application of miRNAs. The aim of this study is therefore to determine the miRNA expression signature of the human fxH in normal fracture healing, and to examine the potential relationship between clinical parameters and these expression levels. MiRNAs can have multiple mRNA targets which affect different cellular processes. Determining these mRNA targets is therefore required to clarify the physiological functions of miRNAs in the different phases of the fracture healing cascade.

## 2 Materials & methods

### 2.1 Primary human samples

Patients with a long bone fracture who were admitted to the division of traumasurgery at the Maastricht University Medical Center (MUMC+) were screened for inclusion in the study. Inclusion criteria were age ≥18 years and a fracture of long bones requiring open reduction and internal fixation (ORIF). Exclusion criteria were pathological fractures, fractures that occurred more than 21 days before fracture surgery, contaminated open fractures, irrigation of the fracture site prior to fxH harvesting and active bleeding at the fracture site if this prohibited accurate fxH harvesting.

To accurately harvest fxH, the surgeon performed this during ORIF fracture surgery according to a standard surgical procedure using a non-traumatic technique. Harvesting fxH was only performed directly from, or near the fracture site; harvesting surrounding tissue, as well as harvesting from the medullary cavity, was avoided since this may introduce other tissue or bone marrow to the sample.

For analysis, the following patient characteristics were obtained: fracture healing outcome (normal/disturbed), gender, age, smoking (pack years), number of days between trauma and primary surgery, fracture occurred during multi-trauma event (yes/no), diabetes mellitus (yes/no), osteoporosis (yes/no), hypertension (yes/no), and arthritic diseases (yes/no). An important parameter in this study is the time interval between trauma and primary surgery, as this allows for miRNA expression analysis at different phases of fracture healing. Normal fracture healing was defined as adequate consolidation based on X-ray imaging and examination throughout a clinical follow up after 6 weeks, 3 and 6 months.

This study was approved by the local ethical committee of the MUMC+ (approval number: METC 16-4-251). The study was performed according to the Declaration of Helsinki in its most recent version.

### 2.2 Sample processing and miRNA extraction

Immediately after harvesting, the fxH was transferred to 2 ​ml microcentrifuge tubes (Biotix, San Diego, USA) using sterile tweezers after which it was snap frozen in liquid nitrogen. Prior to RNA extraction, the fxH was disrupted by adding Trizol Reagent (Thermo Fisher Scientific, Waltham, USA) and using a Qiagen TissueLyser LT (Qiagen, Venlo, The Netherlands). The homogenate was snap frozen in liquid nitrogen and subsequently lysed for 5 ​min. Trizol Reagent was added in a 0.7:1 ratio, based on fxH sample volume.

RNA extraction was performed by chloroform phenol extraction. The amount and purity of the RNA isolates were analysed by spectrophotometry (Biodrop μLite+, Biochrom, Holliston, USA). For samples to be included in this study, RNA purity cut off values of 1.7 and 1.8 were chosen for the A260/A230 and A260/A280 ratios respectively. Per patient, 250 ​ng template RNA were used for the transcription of miRNA to cDNA using the miScript II RT kit (Qiagen, Venlo, The Netherlands) according to the manufacturer's instructions.

### 2.3 qPCR miRNA arrays

For miRNA array analysis, patient cDNA samples were pooled by using equal amounts of cDNA from each sample. Two arrays for miRNAs were used, that being the inflammatory response and autoimmunity array (MIHS-105ZD-2, Qiagen) and the fibrosis array (MIHS-117ZD-2, Qiagen). The miRNA signature of the pooled sample group was determined by these two qPCR miRNA arrays containing primers for 84 individual miRNAs each, excluding housekeeper genes and controls. Since the two arrays showed overlap with regard to the miRNA primers that they contained, the total amount of miRNAs that were investigated was 145. SNORD61, SNORD68, SNORD72, SNORD95, SNORD96A, RNU6-6p were used as housekeeper genes. Further controls included the miRNA reverse transcription control, positive PCR control and the spike in control cel-miRNA-39-3p.

The expression levels of the miRNAs from the array were determined by the cycle number (Cq) via qPCR using the CFX96 Real-Time PCR system (Bio-Rad, Munich, Germany). A ΔCq ≥2 was chosen as a cut-off value to define up- or downregulation. To assess the quality of the qPCR array reactions, melting curve analyses were performed in combination with a chosen cut off Cq value of 35, above which miRNAs were not considered for further analysis due to minute or absent expression.

From each of the two arrays, the top 5 upregulated and top 5 downregulated miRNAs were selected for further individual validation within the study population. This resulted in a total of 20 miRNAs for further validation in the study population.

### 2.4 miRNA validation analysis

The selected 20 miRNAs were validated in each individual sample. For miRNA validation in the individual samples, SNORD96A was chosen as housekeeper gene since its expression approximated the mean expression of the six housekeeper genes provided in the array the most. The regulated miRNAs and the housekeeper gene were validated in duplicates for all patients using the miScript SYBR Green PCR Kit (Qiagen, Venlo, The Netherlands) and the CFX96 Touch Real-Time PCR system (Bio-Rad, Munich, Germany). The expression of each miRNA was determined by the Cq value, which was normalised to the average of SNORD96A expression by means of the ΔCq method. To assess the quality of the single patient qPCR reaction, melting curve analyses were performed in combination with a chosen cut off Cq value of 38, above which the miRNA was not considered to be present in the sample.

### 2.5 *In silico* miRNA target analysis

Qiagen's Ingenuity Pathway Analysis (IPA) was used to identify miRNA targets of the validated miRNAs, which expression levels showed to be dependent on clinical parameters. The IPA software groups miRNA targets based on known scientific data, level of evidence, and cell & tissue type. For this study, experimentally observed and highly predicted human miRNA targets were incorporated into the analysis. The following cell types were included in the analyses: osteoblasts, chondrocytes, mesenchymal stem cells, stromal cells, fibroblasts, neutrophils, and bone marrow cells.

### 2.6 Statistical analysis

All analyses were performed with GraphPad Prism version 9.1.1 (GraphPad Software, San Diego, USA). Data are presented as mean or number, accompanied by standard deviation or percentage as appropriate. Gender, smoking, age, and number of days between trauma and primary surgery were recorded as continuous variables, the remaining variables were dichotomised.

Descriptive statistics were performed on the obtained patient characteristics and miRNA expression data. Subsequently, a stepwise multiple linear regression model was made to examine potential correlations between miRNA expression and patient characteristics. The following parameters were incorporated: gender, age, smoking (pack years), number of days between trauma and primary surgery, fracture occurred during multi-trauma event, diabetes mellitus, osteoporosis, hypertension, and arthritic diseases.

Multiple linear regression, Mann–Kendall test, two samples t-test, and simple linear regression analyses were applied as appropriate to determine the effect of clinical parameters on miRNA expression. For this study, an α of 0.05 was considered statistically significant.

## 3 Results

### 3.1 Patient demographics

The study population consisted of 61 patients (mean age 52 ​± ​19 years; 32♀). Patients underwent surgery 7 ​± ​5 days after trauma. All patients showed uneventful fracture healing after surgery. Thirty-one percent of patients smoked; no data could be collected on smoking for the remaining 30% of patients. Twenty-one percent of the patient population suffered from a fracture that occurred during a multi-trauma event and 34% of patients suffered from one or multiple comorbidities (Table 1). No significant relation was observed between patient age and the difference in days between trauma and primary surgery.

**Table 1:** Patient demographics. Data are depicted as mean or number, accompanied by standard deviation or percentage as appropriate. ∗ Four patients suffered from multiple comorbidities.

	Included patients **(*n ​= ​61*)**
**Gender n (%)**	
Female	32 (52%)
**Age years (± SD)**	52 ​± ​19
**ΔDays trauma to surgery n (± SD)**	7 ​± ​5
Min - max	0–19
**Smoking n (%)**	
Yes	19 (31%)
*Pack years (± SD)*	*10.2 ​± ​16.3*
No	24 (39%)
Unknown	18 (30%)
**Multitrauma n (%)**	13 (21%)
**Comorbidities n (%)∗**	21 (34%)
Diabetes mellitus	3
Osteoporosis	7
Hypertension	13
Arthritic diseases	4

### 3.2 Identification of differentially expressed miRNAs: pooled qPCR arrays

**Fig. 1:**
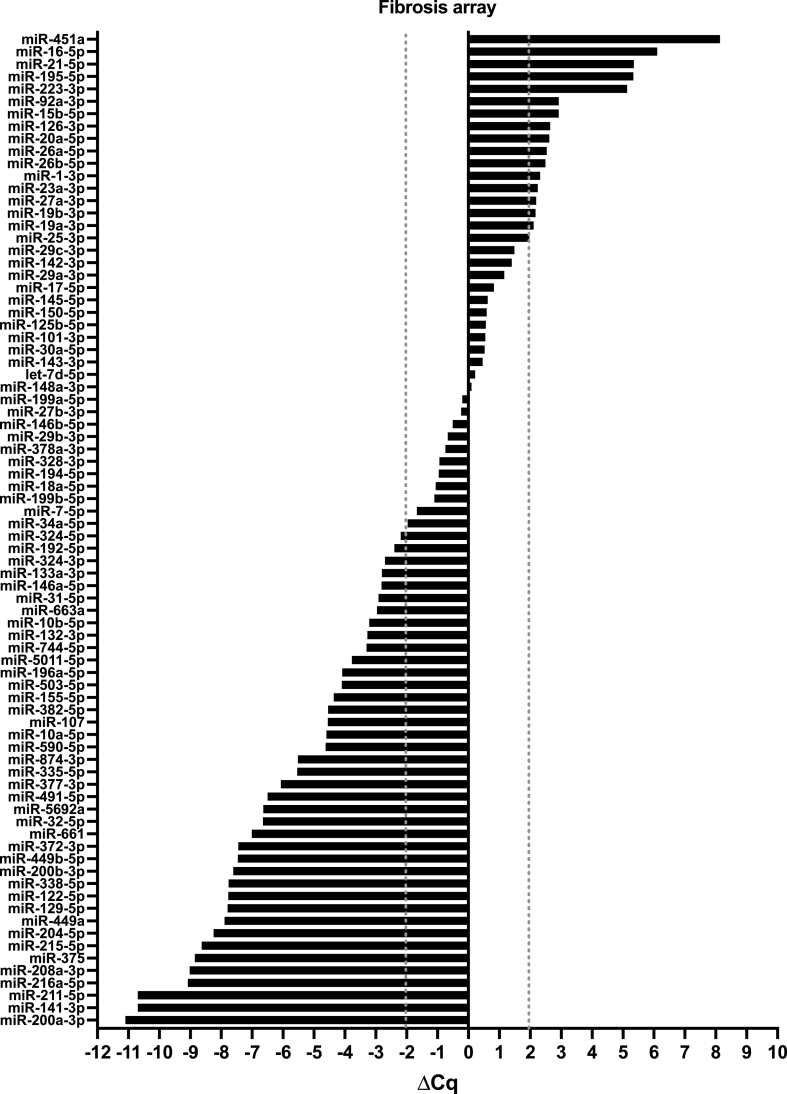
MicroRNA expression signature of pooled fracture hematoma samples (N ​= ​61) in Qiagen's fibrosis array. Results are normalised to the mean of the 6 housekeeper genes and depicted as ΔCq. Grey dotted lines display the threshold of an absolute difference of 2 Cq, normalised to the mean of the housekeeper genes, above or below which microRNAs were considered to be deregulated.

**Fig. 2:**
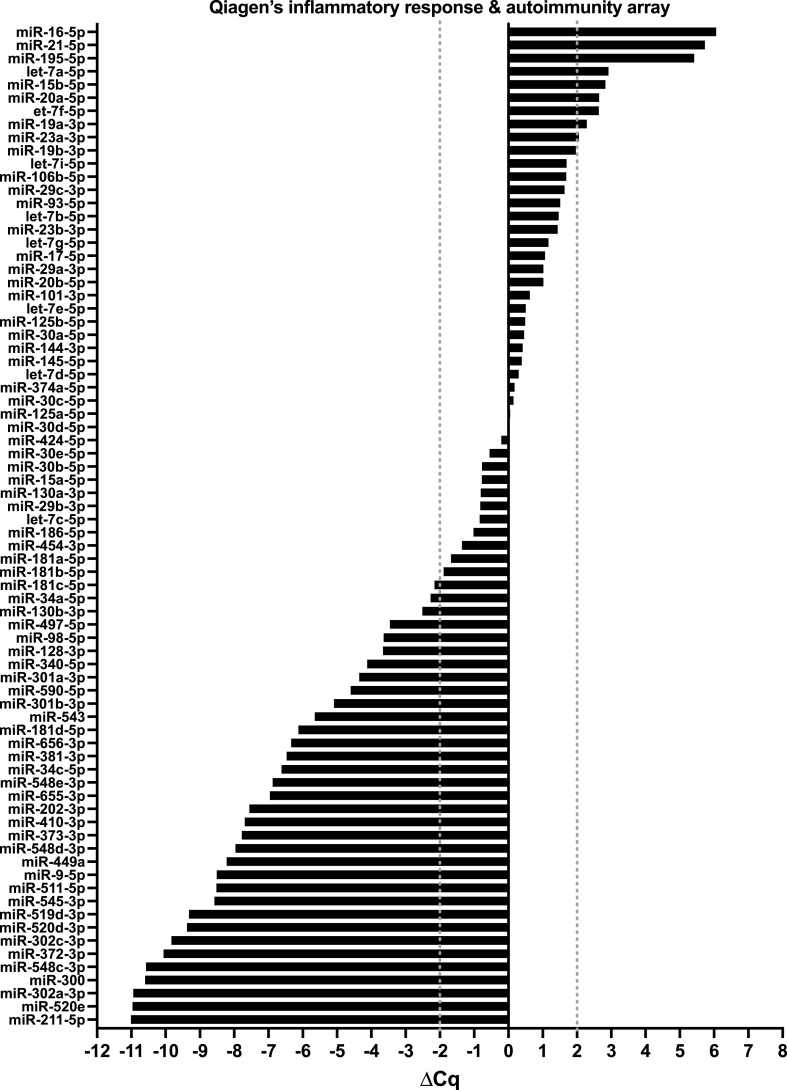
MicroRNA expression signature of the pooled fracture hematoma samples (N ​= ​61) in Qiagen's inflammatory response & auto-immunity array. Results are normalised to the mean of the 6 housekeeper genes and depicted as ΔCq. Grey dotted lines display the threshold of an absolute difference of 2 Cq, normalised to the mean of the housekeeper genes, above- or below which microRNAs were considered to be deregulated.

A total of 145 miRNAs were analysed. In the inflammatory response and autoimmunity array, 76 miRNAs were detected in the fxH samples, out of which 43 were up- or down-regulated (Fig. 1). In the fibrosis array, 80 miRNAs were detected in the fxH samples, out of which 56 were considered up- or down-regulated (Fig. 2).

From each of the two arrays, the top 5 upregulated and top 5 downregulated miRNAs, 20 in total, were selected for further individual validation within the study population (Supplementary Table 1).

### 3.3 miRNA validation

**Fig. 3:**
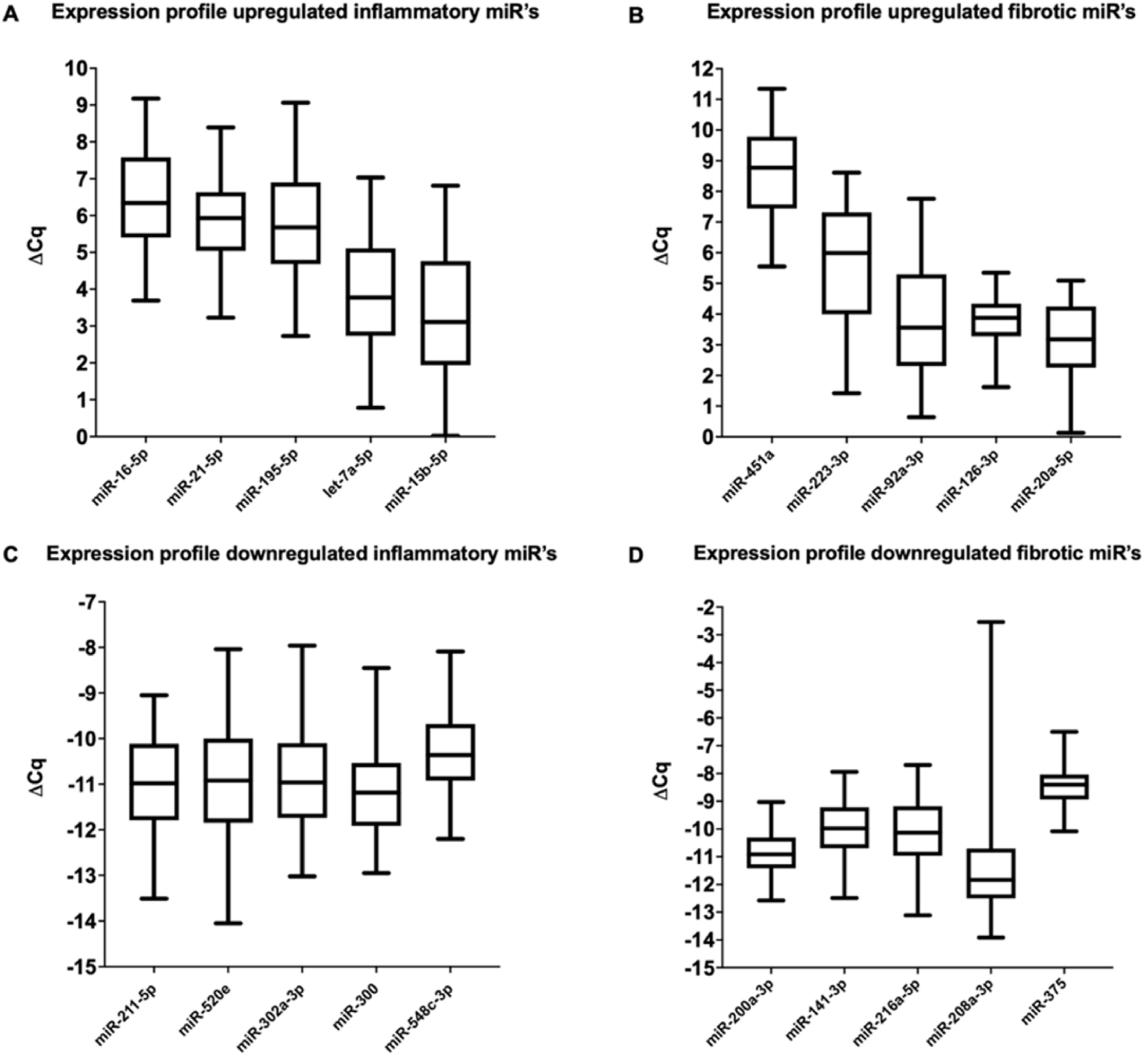
MicroRNAs are differentially expressed in fracture hematoma from patients with normal fracture healing. Box plots show miRNA expression data from the study population validation per category: A) validated top 5 upregulated inflammatory miRNAs; B) validated top 5 downregulated inflammatory miRNAs; C) validated top 5 upregulated fibrotic miRNAs; D) validated top 5 downregulated fibrotic miRNAs. Results are depicted as mean ΔCq. Box plots show the 25th, 50th, and 75th percentiles (horizontal bars) and minimum to maximum ranges (error bars).

Each of the 20 miRNAs was validated in the study population, validation data were consistent with array data (Supplementary Table 1). All 20 miRNAs were up- or downregulated in all individual patients, in line with the results obtained in the arrays (Fig. 3). Each patient showed expression for all of the upregulated miRNAs. Contrarily, not all downregulated miRNAs were expressed in each patient (Supplementary Table 2).

### 3.4 Multiple linear regression model

Multiple linear regression analyses showed that expression levels from seven of the 20 validated miRNAs were dependent on the following clinical parameters: patient age, number of days between trauma and primary surgery, or fracture in multi-trauma. For all miRNAs that showed expression based on patient age, or the number of days between trauma and primary surgery, Mann–Kendall tests showed significant differences (all p ​< ​0.05). Neither comorbidities nor smoking influenced miRNA expression levels in fxH in this study population.

**Fig. 4:**
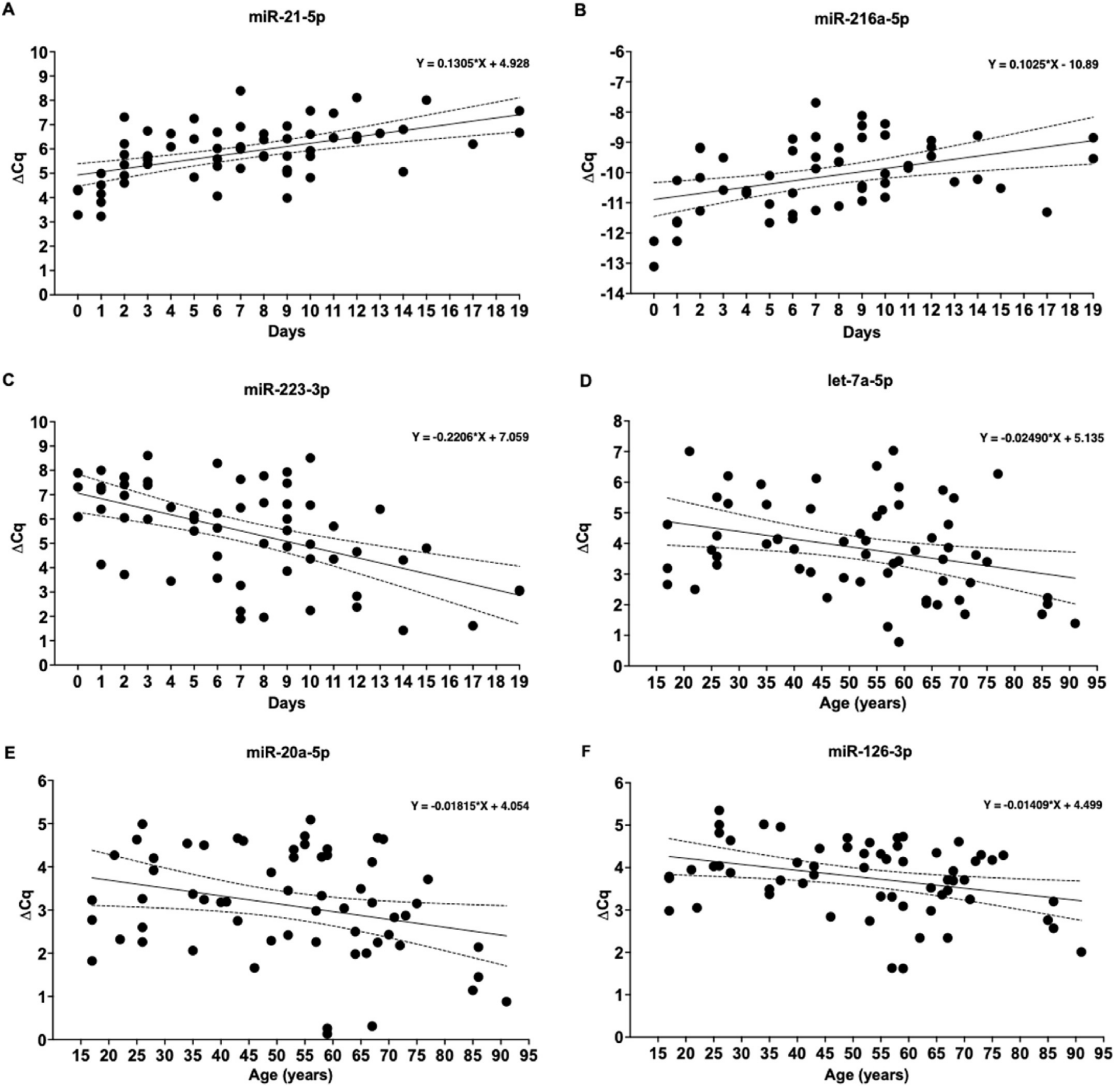
Regression analysis of six miRNAs whose expression showed a significant dependence on either the time interval between trauma and surgery, or patient age. Every dot represents one patient, dotted lines represent 95% confidence intervals. Time-dependent expression of: A) miRNA-21-5p, p ​< ​0.0001; B) miRNA-216a-5p, p ​= ​0.002; C) miRNA-223-3p, p ​< ​0.0001. Age-dependent expression of: D) let-7a-5p, p ​= ​0.013; E) miRNA-20a-5p, p ​= ​0.027; F) miRNA-126-3p, p ​= ​0.011. The regression equation is shown for each graph.

**Fig. 5:**
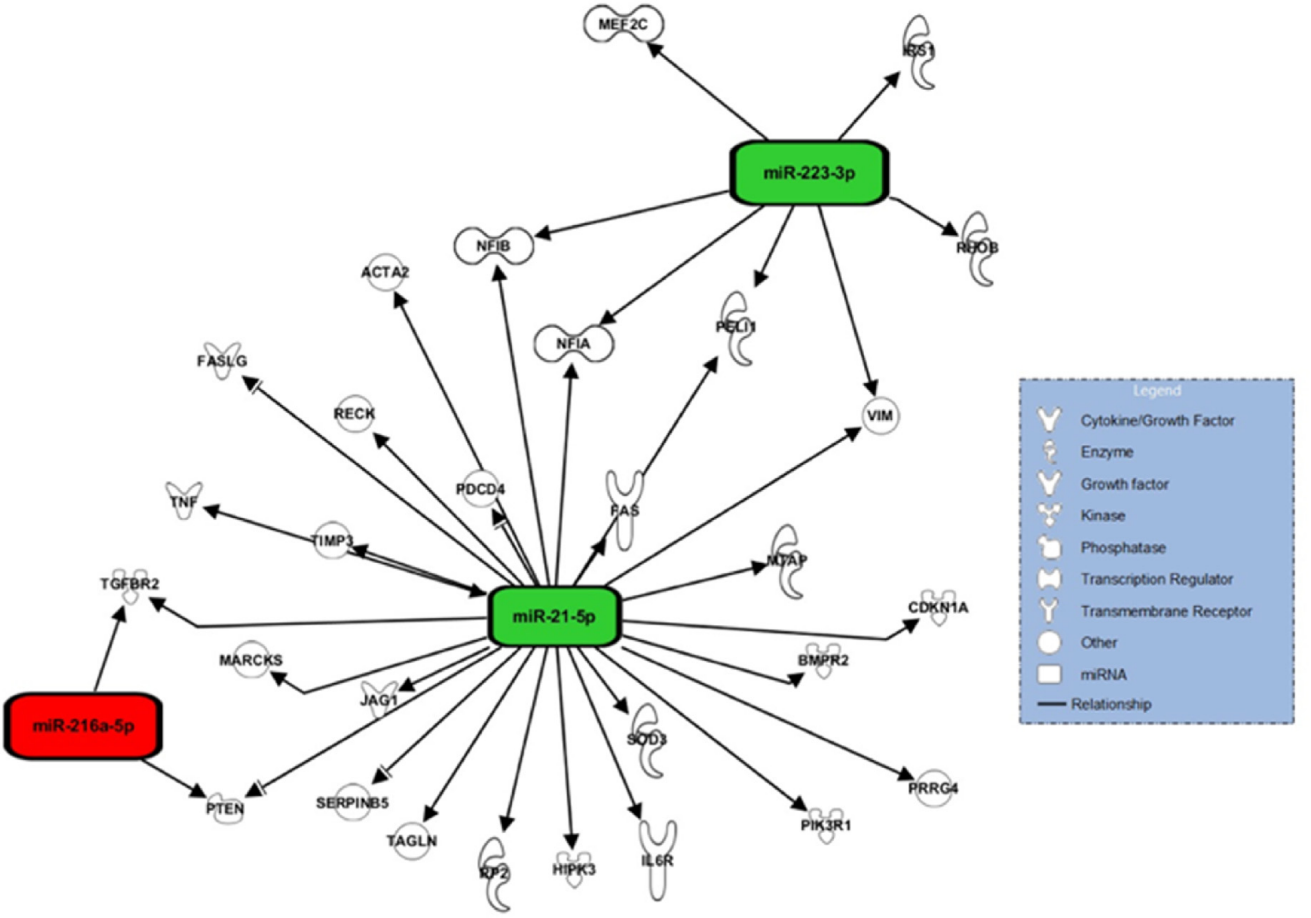
In silico target analysis, performed with Qiagen Ingenuity Pathway Analysis (IPA) software, of 3 miRNAs whose expression was dependent on the time interval between trauma and primary surgery. Green colour represents upregulated miRNA expression, red colour represents downregulated miRNA expression.

For three miRNAs, expression was correlated on the time interval between trauma and primary surgery: miRNA-21-5p, miRNA-216a-5p, and miRNA-223-3p (Fig. 4, Fig. 5). miRNA-21-5p resulted consistently upregulated in all patient samples, and its expression level was higher in patients with longer intervals between trauma and surgery (p ​< ​0.0001; Fig. 4A). Similarly, the downregulated miRNA-216a-5p showed higher expression in patients with longer time intervals between trauma and surgery (p ​= ​0.002; Fig. 4B). On the contrary, the upregulated miRNA-223-3p showed a trend to decrease its expression with longer intervals between trauma and surgery (p ​< ​0.0001; Fig. 4C).

**Fig. 6:**
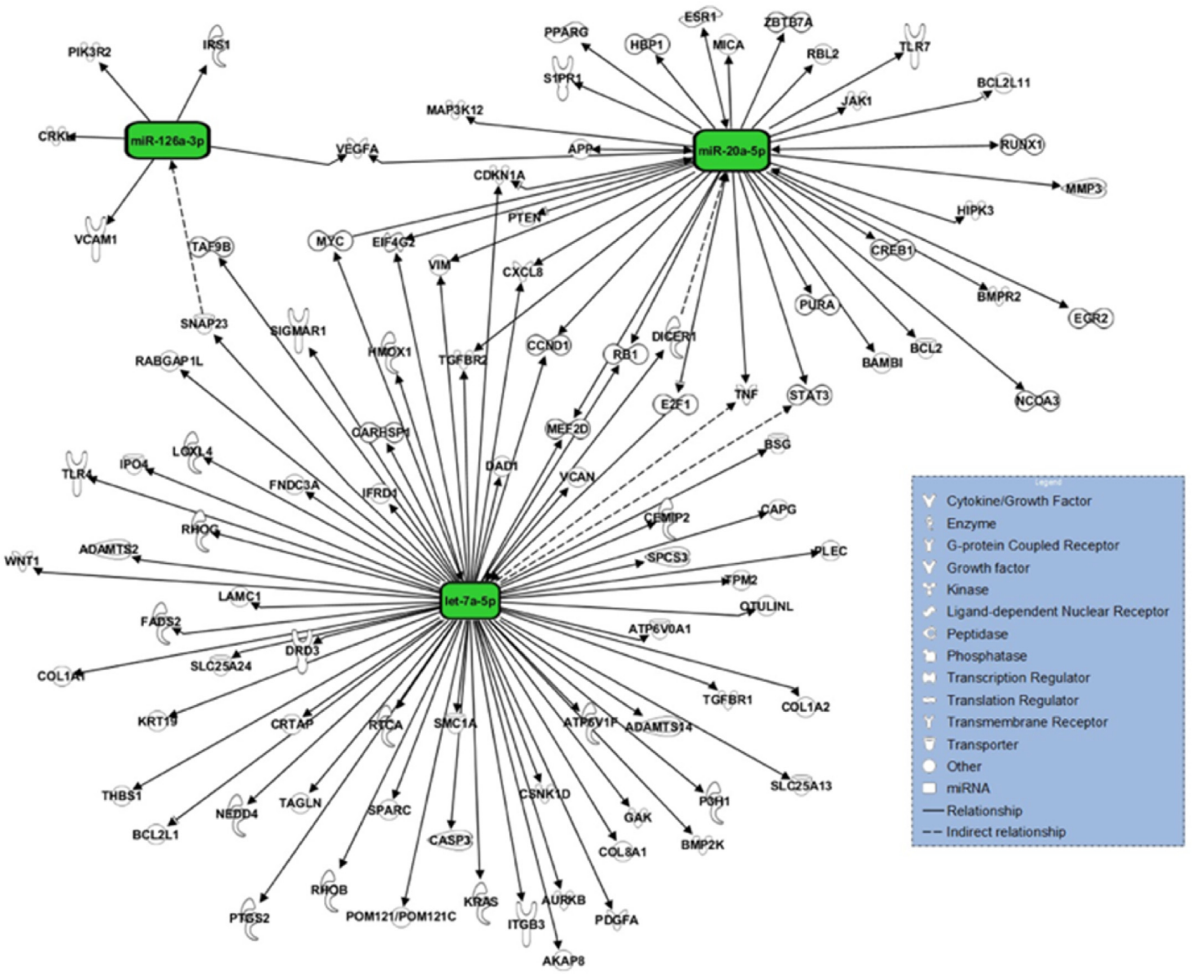
In silico target analysis, performed with Qiagen Ingenuity Pathway Analysis (IPA) software, of 3 miRNAs whose expression was dependent on patient age. Green colour represents upregulated miRNA expression.

Three other miRNAs showed patient age-dependent expression: let-7a-5p, miRNA-20a-5p, and miRNA-126-3p (p ​= ​0.013, p ​= ​0.027, p ​= ​0.011, respectively; Fig. 4, Fig. 6). All three miRNAs were upregulated with a trend to decrease their expression as the patient's age increased.

**Fig. 7:**
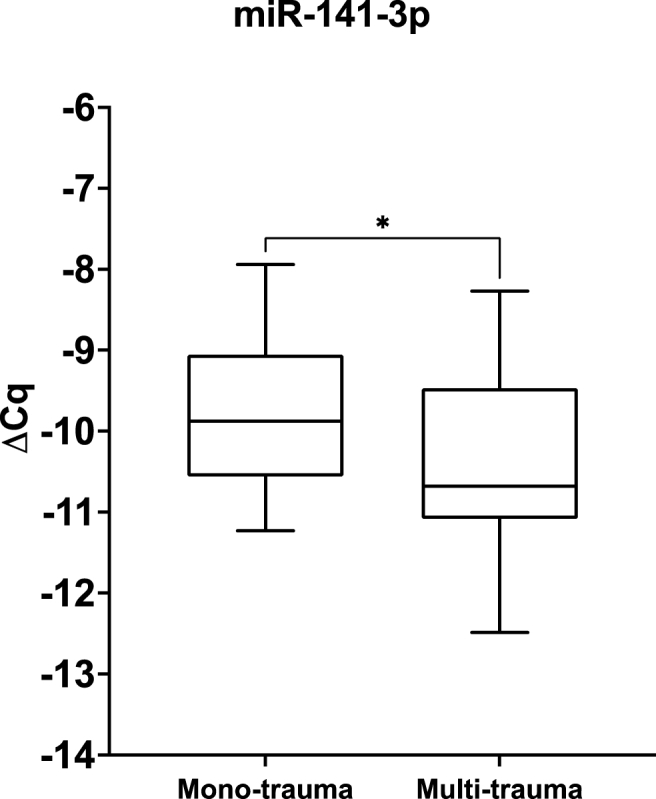
MiRNA-141-3p is differentially expressed in fracture hematoma from patients with normal fracture healing, depending on the severity of trauma. MicroRNA expression data of miRNA-141-3p in patients who suffered from mono-trauma (n ​= ​43) as compared to multi-trauma (n ​= ​12). Results are depicted as mean ΔCq. Box plots show the 25th, 50th, and 75th percentiles (horizontal bars) and minimum to maximum ranges (error bars). ∗p ​= ​0.033.

Lastly, miRNA-141-3p was downregulated and showed lower expression in multi-trauma patients as compared to mono-trauma patients (p ​= ​0.033; Fig. 7).

### 3.5 *In silico* target analysis

Qiagen IPA software identified 260 targets for 14 out of the validated 20 miRNAs. For the seven miRNAs whose expression levels showed to be dependent on a clinical parameter, 124 targets were identified. Several targets involved in various aspects of fracture healing were observed to overlap between the different miRNAs, such as, Phosphatase and Tensin homolog (PTEN), Transforming Growth Factor Beta Receptor 2 (TGFBR2), Tumor Necrosis Factor (TNF), Vimentin (VIM), Nuclear Factor 1A (NFIA), Signal Transducer and Activator of Transcription 3 (STAT3), and Vascular Endothelial Growth Factor A (VEGFA) (Fig. 5, Fig. 6).

## 4 Discussion

To the best of our knowledge, the present study is the first to report on the miRNA involvement in human fxH, correlations between miRNA expression in the human fxH and clinical parameters, and *in silico* target prediction of their mRNA targets in the normal fracture healing cascade. MiRNAs are important regulatory molecules in fracture healing, this study can therefore potentially function as a benchmark for future research on miRNAs in fxH, both in normal as well as impaired fracture healing.

### 4.1 Time dependent miRNA expression

The time interval between trauma and primary surgery showed to influence the expression of three miRNAs, miRNA-21-5p, miRNA-223-3p, and miRNA-216a-5p. In this study, miRNA-21-5p was upregulated and increasingly expressed over time. MiRNA-21-5p is a mechanosensitive, anti-inflammatory miRNA whose expression increases in inflammatory conditions. It has been shown to enhance osteogenic differentiation, angiogenesis, mineralisation, and osteoclastogenesis *in vivo*. This is in line with the results from our *in silico* target analysis which, among others, identified PTEN, TGFBR2, TNF, VIM and NFIA as mRNA targets for miRNA-21-5p [[16], [17], [18], [19], [20]]. Furthermore, miRNA-21-5p plays a role in M2 macrophage polarisation, which results in enhanced regeneration at the fracture site [21]. Kelch et al. found that miRNA-21-5p expression in serum could serve as a potential biomarker for osteoporosis due to its increasing expression over time in non-osteoporotic patients as compared to decreasing expression over time in osteoporotic patients [13]. This is in line with the expression pattern of miRNA-21-5p that was observed in our study for the fxH of patients with normal fracture healing. MiRNA-21-5p expression increased over time. This may be an indication of an existing correlation between miRNA-21-5p expression and the progression of fracture healing with concomitant osteogenesis.

MiRNA-223-3p, upregulated and decreasingly expressed over time, is an important regulator of the immune response. It shows high expression by hematopoietic cells and is involved in, among others, macrophage and neutrophil functioning. It dampens the inflammatory response and, like miRNA-21-5p, enhances M2 macrophage polarisation [22]. Besides regulating inflammation and polarising macrophages towards the more regenerative phenotype, miRNA-223-3p is also likely to stimulate osteoblast differentiation due to the targeting of VIM [19]. Furthermore, it regulates osteoclast functioning by targeting NFIA which normally inhibits the binding of Macrophage Colony Stimulating Factor to its receptor [20].

MiRNA-216a-5p, downregulated and increasingly expressed over time, has shown to enhance osteogenic differentiation and bone formation *in vivo* in osteoporosis [23]. The osteogenic capabilities of miRNA-216a-5p are in part attributable through targeting PTEN and TGFBR2 [14,18]. Furthermore, it mediates the proliferation and migration of chondrocytes whilst inhibiting apoptosis [24]. The expression pattern of miRNA-216a-5p indicates that it may be involved in later phases of the fracture healing cascade and is therefore thought to be involved in osteogenesis.

Putting the expression levels of these three time dependently expressed miRNAs in perspective, it can be stated that the increasing expression of miRNA-21-5p over time matches physiological fracture healing since it dampens the immune response and enhances multiple processes that are involved in osteogenesis. The gradual decline of miRNA-223-3p expression over time could be attributed to the physiological fracture healing cascade in which the inflammatory phase transiently progresses towards a more stable, regenerative phase where soft callus is formed [1]. Lastly, the increasing expression of miRNA-216a-5p over time conforms to chondrocyte activities and osteoblast differentiation during physiological fracture healing. Overall, the expression of the three time dependently expressed miRNAs have shown to be important in regulating fracture healing on a molecular level.

### 4.2 Age dependent miRNA expression

Three miRNAs were expressed in an age dependent manner, all of which were upregulated and higher expressed in younger patients. One of the three, let-7a-5p, is among the first miRNAs discovered and has numerous functions within eukaryotic cells, such as the augmentation of inflammation and apoptosis, whilst reducing cellular proliferation [25]. Interestingly, let-7a-5p seems to play opposing roles in osteoblast functioning and mineralisation. The results from this study suggest that let-7a-5p is beneficial for osteogenesis, since its expression is increased in younger patients with normal fracture healing. Remarkably, it targets TGFBR2, STAT3 and VIM, all three involved in osteogenesis through enhancing osteoprogenitor cell differentiation, mineralisation or angiogenesis [14,19,26].

MiRNA-20a-5p has shown to reduce inflammation and apoptosis, whilst enhancing osteogenic differentiation and modulating angiogenesis [19,27,28]. These characteristics can in part be attributed to the targeting of PTEN, VIM, TNF, STAT3 and VEGFA [16,17]. Furthermore, Retino Blastoma 1 and E2 Transcription Factor 1 were identified as targets of miRNA-20a-5p, indicating that it is involved in cell cycle progression [29].

Lastly, miRNA-126a-3p has shown to promote chondrocyte migration and proliferation whilst reducing inflammation and apoptosis *in vivo* [30]. Furthermore, Phosphoinositide-3-Kinase Regulatory Subunit 2, VEGFA and Vascular Endothelial Adhesion Molecule 1 (VCAM1) were identified as targets of miRNA-126a-3p and are known to regulate angiogenesis [31,32]. However, controversy exists on the angiogenic effects of miRNA-126a-3p. VCAM1, an important cellular adhesion molecule, is also targeted by miRNA-126a-3p. Under normal circumstances, VCAM1 is involved in inflammation by enabling the adherence of immune cells to the vascular endothelium, enabling their extravasation. In recent years, it has also gained increasing attention in oncology due to its angiogenic capabilities [33].

Placing the age dependent decline in expression levels of the above-mentioned miRNAs and their targets into perspective offers novel insights. The pro-inflammatory characteristics and seemingly contradictory effects of let-7a-5p on osteogenesis point out the requirement for more specific research in the field of bone regeneration in order to make solid claims about its function therein. On the other hand, both miRNA-20a-5p and miRNA-126a-3p reduce inflammation and apoptosis whilst enhancing osteogenesis and regulating angiogenesis. However, research on the mechanisms behind this regulation is lacking, underlining the need for future research to examine their angiogenic characteristics in the context of fracture healing.

### 4.3 Multi-trauma dependent expression

The expression of miRNA-141-3p was dependent on the severity of the trauma; mono-vs. multi-trauma. MiRNA-141-3p showed decreased expression in the fxH of multiply traumatised patients as compared to the fxH of mono-trauma patients. The immune system might play a role in this expression pattern since the severity of the trauma greatly influences the immunological response [34]. MiRNA-141-3p has shown to be downregulated in sepsis and regulate the inflammatory response [35]. Although predominantly researched in the fields of oncology and gastroenterology, miRNA-141-3p has shown to dampen inflammation and regulate cell proliferation and apoptosis [[36], [37], [38]]. Great variation among the effects of miR-141-3p exists, depending on the tissue or cell type in which it has been investigated, indicating that it may operate in a tissue/cell specific manner. Sangani et al. examined the role of miR-141 in bone regeneration. It exhibited anti-osteogenic characteristics, reducing osteogenic differentiation, as well as mineralisation, in murine BMSCs by direct targeting of Sodium Dependent Vitamin C transporter [39]. Furthermore, miR-141 has shown to directly target Stromal Cell-Derived Factor 1a, a known potentiator of osteogenic differentiation [40]. The results from the target analysis, which was based on a selection of cells which are involved in osteogenesis, show that miRNA-141-3p targets both PTEN and VIM, confirming that it could possess anti-osteogenic characteristics within the context of fracture healing. Taking the above mentioned into account, it is probable that the decreased expression of miRNA-141-3p is related to a dysregulated immune response as a consequence of trauma severity.

This study has several limitations, the first being a lack of normal control samples. Unfortunately, due to the nature of the samples, controls are inexistent as a fracture hematoma only exists after a fracture occurs. Secondly, the number of patients in some subgroups, such as the different comorbidities, is low. Due to the prevalence, and multi-faceted pathophysiology of impaired fracture healing, investigating the influence of altered miRNA expression on cellular pathways in fracture healing disorders will require a larger sample size.

This study is amongst the first to investigate the expression of miRNAs in patients on this scale and instigates to further investigate the pathophysiology of impaired fracture healing. Collaborations between different research centers can be of great importance therein to enable a more rapid gathering of samples from different disease categories/conditions. Moreover, to accurately map potential tissue specific microRNA expression, it is important to research their expression in other tissues related to fracture healing, such as bone itself, periosteum, or soft tissues surrounding a fracture site. Finally, we believe that the *in vitro* application of microRNAs in both 2D, as well as 3D models, is required to gain more insights into their involvement in biological pathways.

## 5 Conclusion

In conclusion, this study profiled the miRNA expression signature of the human fxH in normal fracture healing and examined the potential relationship with clinical parameters on these expression levels. Eighty-eight miRNAs showed to be up- or downregulated. The expression levels of seven out of the twenty validated (most regulated) miRNAs were correlated to the number of days between trauma and surgery, as well as to patient age or the severity of trauma.

*In silico* target analysis revealed 124 mRNA targets for these seven miRNAs, which all showed to be involved in multiple processes that are important in the fracture healing cascade, such as angiogenesis, mineralisation, and cellular differentiation. Furthermore, several miRNAs showed to influence the same mRNA targets and can therefore operate in a synergistic manner. This study portrayed the regulatory role and importance of miRNAs in fracture healing. Furthermore, this study may serve as a reference for future studies that investigate miRNA expression in impaired fracture healing, that is a non- or poorly healing patient cohort with possible infection, chronic inflammation, poor vascularisation, osteoporosis, high dose corticosteroid treatment, diabetes, or other comorbidities present. Future research should therefore focus on how aberrant miRNA expression could be correlated to impaired fracture healing, for which larger cohorts are necessary due to the lower prevalence of this condition. This should also be associated with concomitant changes in target mRNA expression and protein translation. Molecular mechanisms that have been elucidated in this study can serve as a means for future research in the field of fxH.

## Funding

N.A.

## Declaration of competing interest

There are no conflicts of interest to declare.

## References

[1] ElHawary H.B.A., Abi-Rafeh J., Vorstenbosch J., Xu L., Efanov J.I. (2021). Bone healing and inflammation: principles of fracture and repair. Semin Plast Surg.

[2] Schell H., Duda G.N., Peters A., Tsitsilonis S., Johnson K.A., Schmidt-Bleek K. (2017). The haematoma and its role in bone healing. J Exp Orthop.

[3] Grosso A., Burger M.G., Lunger A., Schaefer D.J., Banfi A., Di Maggio N. (2017). It takes two to tango: coupling of angiogenesis and osteogenesis for bone regeneration. Front Bioeng Biotechnol.

[4] Volpin G.S.H., Bentley G.E. (2014). European surgical orthopaedics and traumatology.

[5] O'Brien J., Hayder H., Zayed Y., Peng C. (2018). Overview of MicroRNA biogenesis, mechanisms of actions, and circulation. Front Endocrinol.

[6] Cui M., Wang H., Yao X., Zhang D., Xie Y., Cui R. (2019). Circulating MicroRNAs in cancer: potential and challenge. Front Genet.

[7] Kim M., Zhang X. (2019). The profiling and role of miRNAs in diabetes mellitus. J Diabetes Clin Res.

[8] Fröhlich L.F. (2019). Micrornas at the interface between osteogenesis and angiogenesis as targets for bone regeneration. Cells.

[9] Peng S., Gao D., Gao C., Wei P., Niu M., Shuai C. (2016). MicroRNAs regulate signaling pathways in osteogenic differentiation of mesenchymal stem cells (Review). Mol Med Rep.

[10] Seeliger C., Karpinski K., Haug A.T., Vester H., Schmitt A., Bauer J.S. (2014). Five freely circulating miRNAs and bone tissue miRNAs are associated with osteoporotic fractures. J Bone Miner Res.

[11] Seeliger C., Balmayor E.R., van Griensven M. (2016). miRNAs related to skeletal diseases. Stem Cell Dev.

[12] Geng Z., Yu Y., Li Z., Ma L., Zhu S., Liang Y. (2020). miR-21 promotes osseointegration and mineralization through enhancing both osteogenic and osteoclastic expression. Mater Sci Eng C Mater Biol Appl.

[13] Kelch S., Balmayor E.R., Seeliger C., Vester H., Kirschke J.S., van Griensven M. (2017). miRNAs in bone tissue correlate to bone mineral density and circulating miRNAs are gender independent in osteoporotic patients. Sci Rep.

[14] Wu M., Chen G., Li Y.-P. (2016). TGF-β and BMP signaling in osteoblast, skeletal development, and bone formation, homeostasis and disease. Bone Research.

[15] Nugent M. (2017). MicroRNAs and fracture healing. Calcif Tissue Int.

[16] Li F.-S., Li P.-P., Li L., Deng Y., Hu Y., He B.-C. (2021). PTEN reduces BMP9-induced osteogenic differentiation through inhibiting Wnt10b in mesenchymal stem cells. Front Cell Dev Biol.

[17] Yang C., Liu X., Zhao K., Zhu Y., Hu B., Zhou Y. (2019). miRNA-21 promotes osteogenesis via the PTEN/PI3K/Akt/HIF-1α pathway and enhances bone regeneration in critical size defects. Stem Cell Res Ther.

[18] Kato M., Putta S., Wang M., Yuan H., Lanting L., Nair I. (2009). TGF-beta activates Akt kinase through a microRNA-dependent amplifying circuit targeting PTEN. Nat Cell Biol.

[19] Lian N., Wang W., Li L., Elefteriou F., Yang X. (2009). Vimentin inhibits ATF4-mediated osteocalcin transcription and osteoblast differentiation. J Biol Chem.

[20] Xie Y., Zhang L., Gao Y., Ge W., Tang P. (2015). The multiple roles of microrna-223 in regulating bone metabolism. Molecules.

[21] Song Q., Zhong L., Chen C., Tang Z., Liu H., Zhou Y. (2015). miR-21 synergizes with BMP9 in osteogenic differentiation by activating the BMP9/Smad signaling pathway in murine multilineage cells. Int J Mol Med.

[22] Yuan X., Berg N., Lee J.W., Le T.-T., Neudecker V., Jing N. (2018). MicroRNA miR-223 as regulator of innate immunity. J Leukoc Biol.

[23] Li H., Li T., Fan J., Li T., Fan L., Wang S. (2015). miR-216a rescues dexamethasone suppression of osteogenesis, promotes osteoblast differentiation and enhances bone formation, by regulating c-Cbl-mediated PI3K/AKT pathway. Cell Death Differ.

[24] Rong Y., Zhang J., Jiang D., Ji C., liu W., Wang J. (2021). Hypoxic pretreatment of small extracellular vesicles mediates cartilage repair in osteoarthritis by delivering miR-216a-5p. Acta Biomater.

[25] Sui C., Zhang L., Hu Y. (2019). MicroRNA-let-7a inhibition inhibits LPS-induced inflammatory injury of chondrocytes by targeting IL6R. Mol Med Rep.

[26] Sims N.A. (2020). The JAK1/STAT3/SOCS3 axis in bone development, physiology, and pathology. Exp Mol Med.

[27] Sun M., Zhou X., Chen L., Huang S., Leung V., Wu N. (2016). The regulatory roles of MicroRNAs in bone remodeling and perspectives as biomarkers in osteoporosis. BioMed Res Int.

[28] Yuan J., Su Z., Gu W., Shen X., Zhao Q., Shi L. (2019). MiR-19b and miR-20a suppress apoptosis, promote proliferation and induce tumorigenicity of multiple myeloma cells by targeting PTEN. Cancer Biomarkers.

[29] Alberts B ea (2014).

[30] Zhou Y., Ming J., Li Y., Li B., Deng M., Ma Y. (2021). Exosomes derived from miR-126-3p-overexpressing synovial fibroblasts suppress chondrocyte inflammation and cartilage degradation in a rat model of osteoarthritis. Cell Death Discovery.

[31] Cao D., Mikosz A.M., Ringsby A.J., Anderson K.C., Beatman E.L., Koike K. (2020). MicroRNA-126-3p inhibits angiogenic function of human lung microvascular endothelial cells via LAT1 (L-type Amino acid transporter 1)-mediated mTOR (mammalian target of rapamycin) signaling. Arterioscler Thromb Vasc Biol.

[32] Hong Z., Hong C., Ma B., Wang Q., Zhang X., Li L. (2019). MicroRNA-126-3p inhibits the proliferation, migration, invasion, and angiogenesis of triple-negative breast cancer cells by targeting RGS3. Oncol Rep.

[33] Kong D.-H., Kim Y.K., Kim M.R., Jang J.H., Lee S. (2018). Emerging roles of vascular cell adhesion molecule-1 (VCAM-1) in immunological disorders and cancer. Int J Mol Sci.

[34] Huber-Lang M., Lambris J.D., Ward P.A. (2018). Innate immune responses to trauma. Nat Immunol.

[35] Lin X., Wang Y. (2021). miR-141 is negatively correlated with TLR4 in neonatal sepsis and regulates LPS-induced inflammatory responses in monocytes. Braz J Med Biol Res.

[36] Huang Z., Shi T., Zhou Q., Shi S., Zhao R., Shi H. (2014). miR-141 Regulates colonic leukocytic trafficking by targeting CXCL12β during murine colitis and human Crohn's disease. Gut.

[37] Chen H., Zeng L., Zheng W., Li X., Lin B. (2020). Increased expression of microRNA-141-3p improves necrotizing enterocolitis of neonates through targeting MNX1. Frontiers in Pediatrics.

[38] Gao Y., Feng B., Han S., Zhang K., Chen J., Li C. (2016). The roles of MicroRNA-141 in human cancers: from diagnosis to treatment. Cell Physiol Biochem.

[39] Sangani R., Periyasamy-Thandavan S., Kolhe R., Bhattacharyya M.H., Chutkan N., Hunter M. (2015). MicroRNAs-141 and 200a regulate the SVCT2 transporter in bone marrow stromal cells. Mol Cell Endocrinol.

[40] Periyasamy-Thandavan S., Burke J., Mendhe B., Kondrikova G., Kolhe R., Hunter M. (2019). MicroRNA-141-3p negatively modulates SDF-1 expression in age-dependent pathophysiology of human and murine bone marrow stromal cells. J Gerontol A Biol Sci Med Sci.

