# Supplementary material for: Specific microRNAs are associated with fracture healing phases, patient age and multi-trauma
Source: J Orthop Translat. 2022 Aug 31;37:1–11. doi: 10.1016/j.jot.2022.07.002 (PMC9449672; doi:10.1016/j.jot.2022.07.002)
Supplement: Multimedia component 1 [file mmc1.docx]

## Supplements

Supplementary table 1: Selected 20 miRNAs and the expression data from the arrays (array Cq) and the mean expression data from individual patient validation (mean validation Cq). Expression data from the validation are depicted as mean ± SD.

| ***microRNA*** | ***Array Cq*** | ***Mean validation Cq (± SD)*** | | | ***ΔCq*** | | |  |  |
| --- | --- | --- | --- | --- | --- | --- | --- | --- | --- |
| ***Inflammatory upregulated*** | | |  | | |  | | |  |
| miR-16-5p | 17.42 | 18.3 ± 1.32 | | | 0.88 | | |  |  |
| miR- 21-5p | 17.75 | 19.06 ± 1.74 | | | 1.31 | | |  |  |
| miR-195-5p | 18.06 | 19.09 ± 1.36 | | | 1.03 | | |  |  |
| miR-7a-5p | 20.56 | 21.04 ± 1.28 | | | 0.48 | | |  |  |
| miR-15b-5p | 20.65 | 21.53 ± 1.37 | | | 0.88 | | |  |  |
| ***Inflammatory downregulated*** | | | |  | | |  | | |
| miR-211-5p | 34.39 | 35.41 ± 0.89 | | | 1.02 | | |  |  |
| miR-520e | 34.45 | 35.06 ± 1.29 | | | 0.61 | | |  |  |
| miR-302a-3p | 34.42 | 35.26 ± 0.88 | | | 0.84 | | |  |  |
| miR-300 | 34.08 | 35.23 ± 1.35 | | | 1.15 | | |  |  |
| miR-548c-3p | 34.05 | 34.75 ± 1.14 | | | 0.7 | | |  |  |
| ***Fibrotic upregulated*** | |  | | |  | | |  |  |
| miR-451 | 15.39 | 16.11 ± 1.34 | | | 0.72 | | |  |  |
| miR-223-3p | 18.4 | 19.29 ± 1.46 | | | 0.89 | | |  |  |
| miR-92a-3p | 20.61 | 20.93 ± 1.36 | | | 0.32 | | |  |  |
| miR-126-3p | 20.98 | 20.88 ± 1.20 | | | 0.1 | | |  |  |
| miR-20a-3p | 21 | 21.64 ± 1.51 | | | 0.64 | | |  |  |
| ***Fibrotic downregulated*** | |  | | |  | | |  |  |
| miR-200a-3p | 34.66 | 35.14 ± 1.07 | | | 0.48 | | |  |  |
| miR-141-3p | 34.2 | 34.17 ± 1.56 | | | 0.03 | | |  |  |
| miR-216a-3p | 32.61 | 34.47 ± 1.43 | | | 1.86 | | |  |  |
| miR-208a-3p | 32.55 | 34.71 ± 2.32 | | | 2.16 | | |  |  |
| miR-375 | 32.38 | 32.96 ± 0.94 | | | 0.58 | | |  |  |

Supplementary table 2: Overview of number of patients that showed expression for the validated downregulated miRNAs. Study population consisted out of 61 patients.

|  | *Number of patients with miRNA expression* |
| --- | --- |
| *Inflammatory downregulated* |  |
| miR-211-5p | 53 |
| miR-520e | 44 |
| miR-302a-3p | 43 |
| miR-300 | 42 |
| miR-548c-3p | 55 |
| *Fibrosis downregulated* |  |
| miR-200a-3p | 52 |
| miR-141-3p | 54 |
| miR-216a-5p | 54 |
| miR-208a-3p | 24 |
| miR-375 | 59 |
